# miRNA Regulation of NK Cells Antiviral Response in Children With Severe and/or Recurrent Herpes Simplex Virus Infections

**DOI:** 10.3389/fimmu.2020.589866

**Published:** 2021-01-25

**Authors:** Marzena Lenart, Edyta Działo, Anna Kluczewska, Kazimierz Węglarczyk, Anna Szaflarska, Magdalena Rutkowska-Zapała, Marcin Surmiak, Marek Sanak, Anna Pituch-Noworolska, Maciej Siedlar

**Affiliations:** ^1^Department of Clinical Immunology, Institute of Pediatrics, Jagiellonian University Medical College, Krakow, Poland; ^2^II Department of Internal Medicine, Jagiellonian University Medical College, Krakow, Poland

**Keywords:** miRNA, NK cells, Herpes simplex virus, antiviral response, posttranscriptional regulation

## Abstract

Severe and/or recurrent infection with Herpes simplex virus (HSV) is observed in a large group of patients treated in clinical immunology facilities. Atypical and prolonged HSV infection is the most common clinical manifestation of disturbed NK cell development and functions, yet the molecular basis of these disorders is still largely unknown. Since recent findings indicated the importance of miRNA in regulating NK cell development, maturation and functions, the aim of our study was to investigate miRNA expression pattern in NK cells in patients with severe and/or recurrent infections with HSV and analyze the role of these miRNAs in NK cell antiviral response. As a result, miRNA expression pattern analysis of human best known 754 miRNAs revealed that patients with severe and/or recurrent HSV infection had substantially upregulated expression of four miRNAs: miR-27b, miR-199b, miR-369-3p and miR-491-3p, when compared to healthy controls. Selective inhibition of miR-27b, miR-199b, miR-369-3p and miR-491-3p expression in NK-92 cells resulted in profound upregulation of 4 genes (APOBEC3G, MAP2K3, MAVS and TLR7) and downregulation of 36 genes taking part in antiviral response or associated with signaling pathways of Toll-like receptors (TLR), NOD-like receptors, the retinoic acid-inducible gene I (RIG-I)-like receptors (RLRs) and type I IFN-related response. Additionally, flow cytometry analysis revealed that miR-369-3p and miR-491-3p inhibitors downregulated NK cell intracellular perforin expression, while the expression of granzyme B and IFNγ remained unchanged. Taken together, our study suggests a novel mechanism which may promote recurrence and severity of HSV infection, based on miRNAs-dependent posttranscriptional regulation of genes taking part in antiviral response of human NK cells.

## Introduction

Severe and/or recurrent infections with HSV are observed in a considerable group of patients treated in clinical immunology facilities. Atypical and prolonged HSV infection is the most common clinical manifestation of disturbed antiviral response caused by primary NK cell deficiencies, that results from either reduced/absent number of NK cells or disturbed function of these cells (1–3). Nonetheless, in a large group of patients suffering from severe and/or recurrent HSV infection who exhibit normal NK cell numbers and NK cell cytotoxic activity, the molecular basis of their disorder is still largely unknown.

Infections with Herpes viruses are mainly controlled by natural killer (NK) cells, as a part of innate immunity. NK cells are large, granular lymphocytes and their peripheral blood frequency varies between 5 and 20% (4). NK cells have several surface markers/receptors, such as CD16 (Fc gamma receptor type III), important receptor in antibody-dependent cellular cytotoxicity (ADCC) (5) or CD56, characteristic of NK cells producing large amounts of cytokines (6). NK cells kill infected or neoplastic cells. The lysis of altered cell occurs *via* perforin (Prf) and granzymes, secreted from cells’ lysosomes, or through Fas ligand, tumor necrosis factor (TNF) or TNF-related apoptosis-inducing ligand (7). NK cells can also secrete a number of cytokines and chemokines, such as interferon γ (IFN-γ), crucial in antiviral response; TNF, granulocyte-macrophage colony-stimulating factor (GM-CSF), interleukin 5 (IL-5), IL-13, macrophage inflammatory protein (MIP-1) and RANTES (6). The release of these cytokines activates Th1 response, macrophage-mediated killing of intracellular pathogens and up-regulates major histocompatibility complex (MHC) class I molecules expression. NK cells can also provide a co-stimulatory signal to T and B lymphocytes, through CD40L and OX40L (8).

NK cells play a vital role in the control of a number of human viruses. Several studies demonstrated NK cell-dependent protective effects during infections with coxsackievirus, human immunodeficiency virus (HIV), hepatitis C virus (HCV), influenza virus and poxvirus, but most importantly, Herpes viruses (7). Furthermore, the most common manifestation of NK cell deficiency, both in number and function, is severe, recurrent or disseminated infection with herpesviruses, including HSV, Varicella zoster virus (VZV), cytomegalovirus (CMV), Epstein-Barr virus (EBV), etc (2, 7).

The discovery of miRNAs has revealed a new mechanism of gene expression regulation that affect many biological systems, including mammalian immune system. miRNA are small, single-stranded non-coding RNA that negatively regulate gene expression by inducing their degradation or repress translation of the target protein-coding mRNA (9). They are encoded by genomic DNA, transcribed by RNA polymerase II and processed from their primary transcript by the enzymes Drosha and DiGeorge syndrome critical region gene 8 (DCGR8). Pre-miRNA are then exported from the nucleus by exportin 5, and after reaching cytoplasm, are further processed by Dicer, resulting in the formation of miRNA duplex. The duplex is packaged into RNA-induced silencing complex (RISC), which comprises several proteins, including Argonaute. The miRNA guides the complex to its 3’ UTRs, leading to inhibition of translation or decrease of mRNA stability (9, 10). miRNA themselves are coordinately modulated by different effectors when carrying out basic functions. Studies of miRNA show development- and tissue-specific regulation mechanisms, although a considerable amount of known miRNA are expressed universally in various tissues and species (11). miRNA are regulated at the multiple steps of their biogenesis, by sequence-specific RNA binding proteins and double-strand RNA binding domains, as well as by other mechanisms, including RNA editing or methylation (12). miRNA plays a pivotal role in every aspect of cellular activity, such as differentiation, development, metabolism, proliferation and apoptotic cell death. miRNA is also associated with molecular mechanisms of various human diseases, including viral infections and tumorigenesis. Currently, approximately 2,300 human miRNAs have been discovered (13).

Recent advances highlight the importance of miRNA in regulating NK cell development, maturation and functions (14–18). In mice, Dicer- and Dcgr8-deficient NK cells were significantly impaired in survival and turnover, and had impaired function of ITAM-containing activating cell receptors (17), while Dicer-deficient NK cells exhibited reduced survival and impaired maturation with enhanced degranulation and IFN-γ production in response to cytokines (19). The role of specific miRNAs in NK cells has also been studied. For example, modulation of miR-150 expression levels has a different effect of NK cells development (20). A number of miRNAs has also been implied to be crucial for NK cell functionality. A number of miRNAs, such as hsa-miR-155 and hsa-miR-181, regulate expression of IFN-γ in NK cells (19, 21), while other, i.e. miR-223, miR-27a-5p, miR-150, miR-378, and miR-30e, has been reported to suppress the cytotoxic capabilities of NK cells by repressing perforin or granzyme B (22–25).

Recent studies have also shown that pathogens can influence miRNA regulation of NK cell functions in order to evade immune response. Among herpesviruses, targeted miR-155 deletion in murine NK cells result in dramatically diminished effector activities and reduce memory cell numbers in both lymphoid and non-lymphoid tissues after murine CMV infection (26). However, little is known about a possible association of disturbance of miRNA regulation and severe, prolonged HSV infection.

The aim of the study was to investigate 1) miRNA expression pattern in NK cells in patients with severe and/or recurrent HSV infections, and to describe 2) which miRNAs might be associated with increased susceptibility to prolonged and severe HSV infection, and 3) define the genes associated with antiviral response which expression is altered by studied miRNAs, in order to predict the possible new mechanism of NK cell deficiency leading to propagation of HSV infection.

## Materials and Methods

### Patients and Control Group

Thirty-eight patients with severe and/or recurrent HSV-1 infection, including children after HSV-1 infection of central nervous system or ocular infections (3 girls and 2 boys), children with extensive infections of skin and/or mucosa (12 girls and 22 boys), children with accompanying recurrent VZV infection (1 girl and 2 boys) and thirty-nine healthy children (16 girls and 23 boys) were evaluated in this study. There were 2 patients classified to more than one group (1 patient was in group of orolabial herpes and disseminated skin infection, 1 patient was in group of disseminated skin infections and VZV infections) (**Table 1**).

**Table 1:** Characteristic of patients and control group.

	Orolabial HSV	Disseminated skin HSV	HSV ocular/CNS infection	Accompanying recurrent VZV infection	Control
Number of children	20	14	5	3	39
Age (years ± SD)	7 ± 7	4 ± 3	11 ± 5	7 ± 5	8 ± 6
Sex (F/M)	7/13	5/9	3/2	1/2	16/23
NK numbers (n)					
normal	16	8	4	2	ND
decreased	1	4	0	0	ND
increased	3	2	1	1	ND

ND, no data.

All patients were under supervision of the outpatient units of the Department of Clinical Immunology of the University Children’s Hospital in Krakow. The study was approved by the Bioethical Committee of the Jagiellonian University (KBET/284/B/2013).

### NK Cell Isolation

NK cells were isolated from blood samples obtained from analyzed patients and healthy controls. In brief, peripheral blood mononuclear cells (PBMC) isolated from EDTA-treated whole peripheral blood by the standard Ficoll/Isopaque (Pharmacia, Uppsala, Sweden) density gradient centrifugation. NK cells were isolated from PBMC using MACS technology and a NK cell isolation kit (Miltenyi Biotech), in which NK cells were isolated *via* negative selection. In particular, non-NK cells were labeled with a cocktail of biotin-conjugated antibodies and anti-biotin magnetic beads and were then depleted by passage through a paramagnetic MS Columns (Miltenyi Biotech).

### miRNA Expression Analysis

miRNA expression analysis was performed in NK cell population isolated from patients and healthy controls. In brief, the total RNA, enriched in small RNAs, was extracted from NK cell samples using High Pure miRNA Isolation Kit (Roche, Germany) according to the manufacturer’s protocol. The first strand cDNA was obtained from the total RNA (400 ng) samples with Megaplex™ RT Primers (pool A and B, Applied Biosystems, Foster City, CA). miRNA expression profile analysis was performed using TaqMan Array Human MicroRNA Card Set v3.0 (Applied Biosystems) according to manufacturer’s protocol. Real-time PCR was performed using the QuantStudio 7 System (Applied Biosystems). Data was analyzed using ExpressionSuite version 1 software (Applied Biosystems). The results were shown on volcano plot (p-value vs. fold change), with following settings: fold change boundary 2,0 and p-value boundary 0,05. Volcano plot was performed using GraphPad Prism version 6 software.

The expression of miRNAs that were significantly elevated in patients vs. control group and the differences reached statistical significance were analyzed again, by individual real-time PCRs (TaqMan Gene Expression Assays, Applied Biosystems). Briefly, reverse transcription was performed as described above (Megaplex RT primers pool A or B were used for the selected miRs and U6 as a control) and the PCR reactions were performed in triplicates using the QuantStudio 7 System (Applied Biosystems). The fluorescent signals generated during the informative log-linear phase were used to calculate the relative amount of miRs, while U6 was used as a control for each PCR run. The expression of each miRNA was calculated using 2^−ΔΔCT^ method.

### Cell Cultures

Natural Killer Cell line (NK-92) were originally obtained from ATCC (ATCC, Manassas, USA). Cells were cultured in the Alpha Minimum Essential Medium without ribonucleosides and deoxyribonucleosides but with sodium bicarbonate (MEM, Corning, New York, USA) supplemented with 2mM L-Glutamine (Thermo Fisher Scientific, Waltham, USA), 0.2mM myo-Inositol (Sigma-Aldrich, Taufkirchen, Germany), 50mM β-mercaptoethanol (Sigma-Aldrich, Taufkirchen, Germany), 0.02mM Folic Acid (Sigma-Aldrich, Taufkirchen, Germany), 150U/ml recombinant human Il-2 (Thermo Fisher Scientific, Waltham, USA), 12.5% Horse Serum (Thermo Fisher Scientific, Waltham, USA) and 12.5% foetal bovine serum (EURx, Gdansk, Poland). Cells were passaged 24h before all experiments using standard protocol to a 24-well plate.

### Transfection With miRNA Inhibitors

NK-92 cells were transfected with the 500nM of 3’-6-fluorescein (6-FAM) conjugated LNA anti-miR-27b, anti-miR-199b, anti-miR-369-3p, and anti-miR-491-3p (miRCURY LNA miRNA Power Inhibitor, 3’-FAM conjugated, Exiqon) without transfection reagent. Alternatively, cells were transfected with 500nM negative control miR or in the absence of miR inhibitors. After 48h of transfection cells were centrifuged at 200g for 5 min, suspended in PBS and sorted as described below. After sorting, cells were seeded into 24-well plate for 24h and then stimulated with 0.2µg/mL phorbol 12-myristate 13-acetate (PMA, Sigma-Aldrich, Taufkirchen, Germany), 0.5µg/mL Ionomycin (Sigma-Aldrich, Taufkirchen, Germany) and 50ng/mL IL-12 (Fisher Scientific, Waltham, USA) for 3h. Next, cells were harvested and RNA isolation was performed, as described below.

### Cell Sorting

The NK-92 cells transfected with miRNA inhibitors were suspended in phosphate-buffered saline buffer (PBS) and sorted using the FACSAria II cell sorter (BD Biosciences, San Jose, CA, USA). The cells were sorted to obtain FAM- miRNA inhibitors-transfected cells. Sorter was equipped with 488 nm laser for excitation of FITC and PE. The following band-pass filters were used for the measurement of fluorescence (wavelength in nm/power in mW): 530/30 for FITC and 582/15 for PE. Data was acquired and analyzed using BD FACSDiva™ Software (Becton Dickinson). Sorting was performed using 85 µm nozzle with the sheath pressure set at 45 PSI and a flow rate dependent on the concentration of cells, at the sort rate of less than 3600 events/s, using the sort precision mode set for “Good Purity” to obtain pure (usually 97–98%) subpopulations. Sorted cells were collected into FBS-coated polypropylene tubes (BD Biosciences).

### Antiviral Response Gene Expression Analysis

Total RNA was extracted from cell suspension using GenElute™ Total RNA Purification Kit (Sigma Aldrich), then cDNA was generated with the iScript Advanced cDNA synthesis kit (Biorad, Carlsbad CA). For antiviral response gene expression analysis, the PrimePCR predesigned plates (384-well format, Bio-Rad) were used, altogether with SsoAdvanced SYBR Green supermix (Bio-Rad), according to manufacturer’s protocol. Real-time PCR reaction was performed on QuantStudio 7 System. Data analysis was performed with PrimePCR Analysis Software V1.0 (Bio-Rad). The expression of each gene was presented as relative expression normalized to GAPDH, which presented the lowest expression variability.

### Flow Cytometry

Intracellular staining of granzyme B (GrmzB), perforin (Prf) and IFNγ was performed in NK-92 cells transfected with miRNA inhibitors as described above. NK-92 cells were stimulated for 6h with 0,2µg/mL PMA (Sigma-Aldrich, Taufkirchen, Germany), 0.5µg/mL Ionomycin (Sigma-Aldrich, Taufkirchen, Germany), 50ng/mL IL-12 (Fisher Scientific, Waltham, USA) in the presence of BD Golgi Stop (Biosciences, Heidelberg, Germany) according to manufacturer’s instructions. Cells were then washed with PBS, fixed/permeabilized with Cytofix/Cytoperm (BD Biosciences) reagent (20 min, 4°C) and washed twice with Perm/Wash solution (BD). Then, mAbs for intracellular staining were added and the samples were incubated for 30 min at 4°C. The following mouse monoclonal antibodies (mAbs) against human molecules were used: anti-GrzmB PE (clone GB11, Affymetrix, San Diego, CA), anti-Prf PE (clone delta G9, BD Bioscience Pharmigen) and anti-IFNγ APC (clone 25723.11, BD FastImmune). Afterwards, the cells were washed twice in Perm/Wash solution and resuspended in PBS for flow cytometry analysis, which was performed using FACSCanto10 flow cytometer (Becton Dickinson Immunocytometry Systems, Palo Alto, CA).

For all staining experiments, appropriate isotype-matched controls were included. The cells that were positively transfected with the inhibitors were determined on the basis of FAM expression. Data analysis was performed using FlowJo software (Tree Star, Inc, Ashland, OR). The expression of analyzed receptors was assessed and presented as a percentage and median fluorescent intensity (MFI).

### Statistical Analysis

Statistical analysis was performed using GraphPad Prism version 6 (GraphPad Software Inc., San Diego, CA). The normal distribution of values was verified using Shapiro-Wilk test. A non-parametric Kruskal-Wallis test with Dunn’s post-hoc test was used and median ± interquartile range (IQR: Q1—25%, Q3—75%) was shown. The P values <0.05 were considered significant.

## Results

### miRNA Expression Pattern Analysis in NK Cells

**Figure 1:**
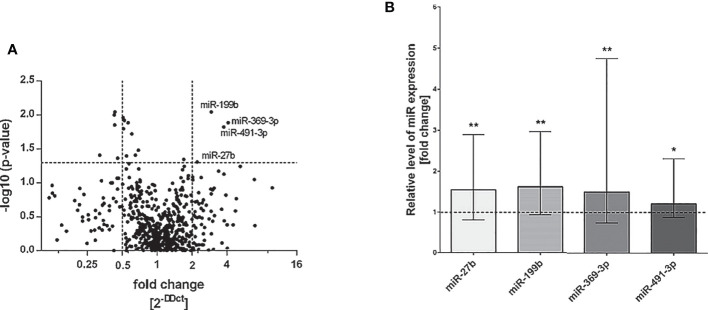
miRNA real-time PCR analysis. Volcano plot showing differential miRNA expression levels in patients with HSV infection in comparison to control group. The vertical lines correspond to 2-fold up and down change and the horizontal line represents a P value of 0,05 **(A)**. Expression of miRNAs miR-27b, miR-199b, miR-369-3p and miR-491-3p presented as fold change of each miR relative expression normalized to U6 expression and healthy control group (2^−ΔΔCT^). The results were obtained from individual real-time PCR reactions performed in all patients and control subjects. Dashed line signifies the control group. Data was analyzed using non-parametric Kruskal-Wallis test with Dunn’s post-hoc test. Asterisks mark significant differences: *p<0,05, **p<0,01 **(B)**.

To evaluate the miRNAs expression pattern in NK cells of patients with severe and/or recurrent HSV infection and healthy control subjects, we performed real-time PCR quantification of the best known 754 human miRNAs in NK cells isolated from peripheral blood of all analyzed individuals. As a result, we noticed nine miRNAs that differed between patient and control group (fold change of ΔCt values of the miRNAs was higher than 2) and the difference was statistically significant (**Figure 1**), where the expression of four miRNA was significantly upregulated in patients group and five were significantly downregulated in patient group (miR-99b, miR-181c, miR-519e, miR-500, miR-552), when compared to healthy controls (**Figure 1A**). Since here we decided to focus on miRNAs which expression was significantly elevated in patient group, we performed individual real-time PCR reactions for these four miRNAs (miR-27b, miR-199b, miR-369-3p, and miR-491-3p) in all patients and healthy subjects, in order to confirm primary results. Again, the differences of analyzed miRNAs expression between patients and control group reached statistical significance (**Figure 1B**). These miRNAs were thus chosen for further analysis.

### The Effect of miRNA Inhibition on Antiviral Gene Expression in NK-92

**Figure 2:**
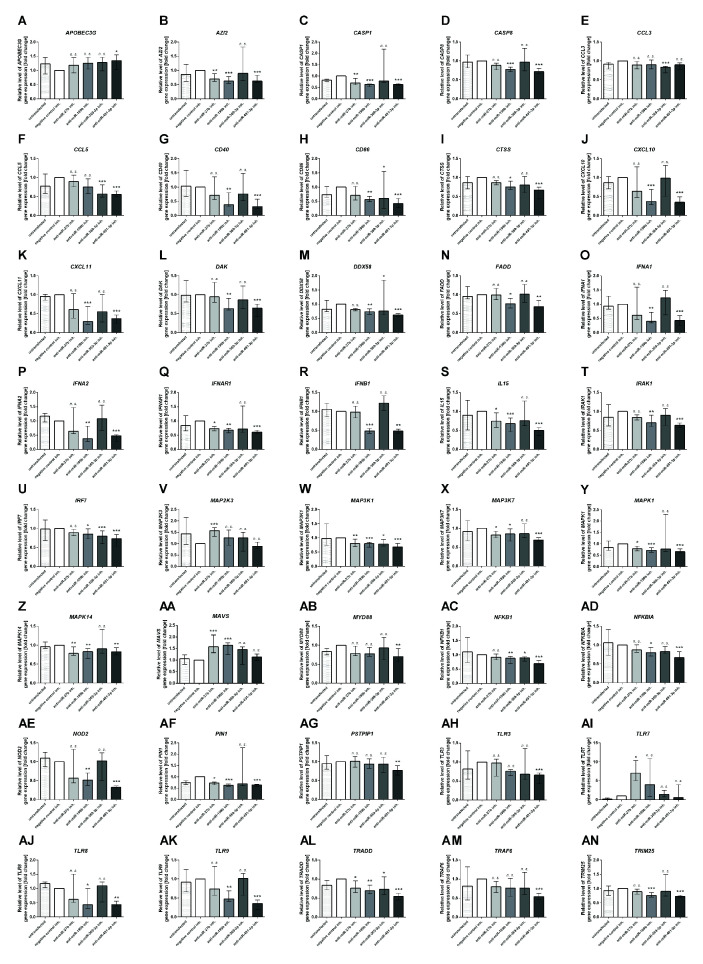
Antiviral gene expression analysis. The effect of miR-27b, miR-199b, miR-369-3p and miR-491-3p inhibition in NK-92 cell line on antiviral response gene expression analyzed by real-time PCR presented as relative expression normalized to GAPDH (2^−ΔΔCT^). Only genes which expression was significantly altered by at least one of the inhibitors are presented on the plot. *APOBEC3G* **(A)**, *AZI2* **(B)**, *CASP1* **(C)**, *CASP8* **(D)**, CCL3 **(E)**, *CCL5* **(F)**, *CD40* **(G)**, *CD86* **(H)**, *CTSS* **(I)**, *CXCL10* **(J)**, *CXCL11* **(K)**, *DAK* **(L)**, *DDX58* **(M)**, *FADD* **(N)**, *IFNA1* **(O)**, *IFNA2* **(P)**, *IFNAR1* **(Q)**, *IFNB1* **(R)**, *IL15* **(S)**, *IRAK1* **(T)**, *IRF7* **(U)**, *MAP2K3* **(V)**, *MAP3K1* **(W)**, *MAP3K7* **(X)**, *MAPK1* **(Y)**, *MAPK14* **(Z)**, *MAVS* **(AA)**, *MYD88* **(AB)**, *NFKB1* **(AC)**, *NOD2* **(AE)**, *PIN1* **(AF)**, *PSTPIP1* **(AG)**, *TLR3* **(AH)**, *TLR7* **(AI)**, *TLR8* **(AJ)**, *TLR9* **(AK)**, *TRADD* **(AL)**, *TRAF6* **(AM)**, *TRIM25* **(AN)**. Plots are presented in an alphabetic order. The data of the effect of each specific miRNA inhibitor was compared to negative control inhibitor. Data was analyzed using non-parametric Kruskal-Wallis test with Dunn’s post-hoc test. Data from 3 independent experiments, median and interquartile range are shown. Asterisks mark significant differences: *p < 0,05, **p < 0,01, ***p < 0,001. inh., inhibitor; n. s., not significant.

**Figure 3:**
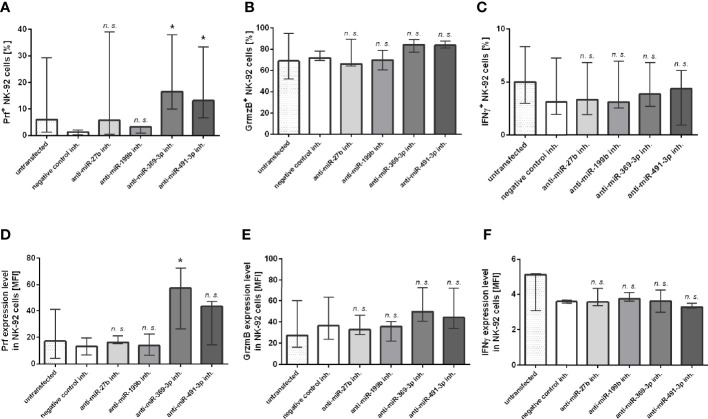
Flow cytometry analysis. The effect of miR-27b, miR-199b, miR-369-3p and miR-491-3p inhibition in NK-92 cell line on perforin, granzyme B and IFNγ expression analyzed using flow cytometry. The percentage of cells expressing perforin **(A)**, granzyme B **(B)** and IFNγ **(C)**, and MFI of perforin **(D)**, granzyme B **(E)** and IFNγ **(F)** is shown. The data of the effect of each specific miRNA inhibitor was compared to negative control inhibitor. Data was analyzed using non-parametric Kruskal-Wallis test with Dunn’s post-hoc test for multiple comparisons. Data from 5 independent experiments, median and interquartile range are shown. Asterisks mark significant differences: *p < 0,05. inh., inhibitor; n. s., not significant.

Next, we focused on the analysis of the role of selected miRNAs in NK cells. Therefore, we performed functional inhibition of each of the selected miRNAs in NK-92 cell line using specific mRNAs inhibitors. NK-92 cells were transfected with anti-miR-27b, anti-miR-199b, anti-miR-369-3p, and anti-miR-491-3p inhibitors, simultaneously with a negative control inhibitor. Preliminary experiments showed that transfection efficacy of NK-92 cells with the inhibitors was low (app. 20%) (data not shown) thus we decided to use the inhibitors that are conjugated with 6-FAM which can be detected by flow cytometry, and isolate positively transfected cells by FACS sorting. The cells viability after transfection was analyzed during sorting on the basis of FSC and SSC parameters, thus only living cells were gated. All gated cells were 6-FAM-positive thus the transfection efficacy was nearly 100% (**Supplementary Figure 1**) and similar in all applied miRNA inhibitors. The cells were than seeded, cultured overnight and stimulated with PMA, Ionomycin and IL-12. Then, real-time PCR was performed in order to determine the expression of 86 genes associated with antiviral response using predesigned real-time PCR plates. The results were presented as a relative expression after normalization to GAPDH. In summary, 4 genes associated with antiviral response were significantly upregulated and 36 downregulated in NK-92 cells by at least one of the miRNA inhibitor, when compared to negative control inhibitor (**Table 2** and **Supplementary Figure S1**). In particular, miR-27b inhibitor significantly upregulated *MAP2K3* (**Figure 2V**), *MAVS* (**Figure 2AA**) and *TLR7* (**Figure 2AI**) genes, and downregulated *IRAK-1* (**Figure 2T**) and *MAPK14* (**Figure 2Z**) gene expression. Anti-miR-199b inhibitor significantly upregulated *MAVS* (**Figure 2AA**) gene and downregulated *AZI2* (**Figure 2B**), *CASP1* (**Figure 2C**), *CASP8* (**Figure 3D**), *CD40* (**Figure 2G**), *CTSS* (**Figure 2I**), *CXCL10* (**Figure 2J**), *CXCL11* (**Figure 2K**), *DAK* (**Figure 2L**), *FADD* (**Figure 2N**), *IFNA1* (**Figure 2O**), *IFNA2* (**Figure 2P**), *IFNAR1* (**Figure 2Q**), *IFNB1* (**Figure 2R**), *MAP3K1* (**Figure 2W**), *MAPK1* (**Figure 2Y**), *MAPK14* (**Figure 2Z**), *NOD2* (**Figure 2AE**), *PIN* (**Figure 2AF**), *TLR8* (**Figure 2AJ**), *TLR9* (**Figure 2AK**), and *TRIM25* (**Figure 2AN**) gene expression. Anti-miR-369-3p inhibitor significantly downregulated *CCL3* (**Figure 2E**), *CCL5* (**Figure 2F**), *CTSS* (**Figure 2I**), *CXCL11* (**Figure 2K**), and *IRF7* (**Figure 2U**) gene expression. Anti-miR-491-3p inhibitor significantly upregulated *APOBEC3G* (**Figure 2A**) and downregulated *AZI2* (**Figure 2B**), *CASP1* (**Figure 2C**), *CASP8* (**Figure 2D**), *CCL5* (**Figure 2F**), *CD40* (**Figure 2G**), *CD86* (**Figure 2H**), *CTSS* (**Figure 2I**), *CXCL10* (**Figure 2J**), *CXCL11* (**Figure 2K**), *DAK* (**Figure 2L**), *DDX58M* (**Figure 2M**), *FADD* (**Figure 2N**), *IFNA1* (**Figure 2O**), *IFNA2* (**Figure 2P**), *IFNAR1* (**Figure 2Q**), *IFNB1* (**Figure 2R**), *IL15* (**Figure 2S**), *IRAK1* (**Figure 2T**), *IRF7* (**Figure 2U**), *MAP3K1* (**Figure 2W**), *MAP3K7* (**Figure 2X**), *MAPK1* (**Figure 2Y**), *MAPK14* (**Figure 2Z**), *MYD88* (**Figure 2AB**), *NFKB1* (**Figure 2AC**), *NFKBIA* (**Figure 2AD**), *NOD2* (**Figure 2AE**), *PIN* (**Figure 2AF**), *PSTPIP1* (**Figure 2AG**), *TLR3* (**Figure 2AH**), *TLR8* (**Figure 2AJ**), *TLR9* (**Figure 2AK**), *TRADD* (**Figure 2AL**), *TRAF6* (**Figure 2AM**), and *TRIM25* (**Figure 2AN**) gene expression. The expression of following genes was not altered by miRNA inhibitors: *ACTB, AIM2, ATG5, CARD9, CASP10, CD80, CHUK, CTSB, CTSL1, CYLD, DHX58, FOS, HSP90AA1, IFIH1, IKBKB, IL12A, IL1B, IL6, IL8, IRF3, IRF5, ISG15, JUN, MAP2K1, MAPK3, MAPK8, MEFV, MX1, NLRP3, OAS2, PYCARD, PYDC1, RIPK1, RPLP0, SPP1, STAT1, SUGT1, TBK1, TICAM1, TNF*, and *TRAF3*, when compared to negative control inhibitor. Four genes were undetermined: *CXCL9, DDX3X, IL12b, IL18*.

**Table 2:** Expression of antiviral response genes significantly altered in NK-92 cell line treated with each miRNA inhibitor in comparison to the negative control inhibitor.

miRNA inhibitor	Up-/down- regulation	TLR signaling and responsive genes	NOD-like receptor signaling and responsive genes	(RIG-I)-like receptors signaling and responsive genes	type I IFN signaling and responsive genes
miR-27b	Up	*TLR7, MAP2K3*	*n. d*.	*MAVS*	*n. d*.
	Down	*MAP3K7, MAPK1, MAPK14*	*n. d*.	*AZI2, CASP1, MAP3K1, PIN1, TRADD*	*IFNAR1, IL15*,
miR-199b	Up	*n. d*.	*n. d*.	*MAVS*	*n. d*.
	Down	*CD40, CD86, CTSS, CXCL10, CXCL11, IFNA1, IFNA2, IFNB1, IRF7, MAP3K7, MAPK1, MAPK14, NFKB1, NFKBIA, TLR8, TLR9*	*CASP1, NOD2*,	*AZI2, CASP8, CXCL10, DAK, DDX58, FADD, MAP3K1, MAPK14, PIN1, TRADD, TRIM25*	*IFNA1, IFNA2, IFNAR1, IFNB1, IL15*,
miR-369-3p	Up	*n. d*.	*n. d*.	*n. d*.	*n. d*.
	Down	*CCL3, CCL5, CTSS, IRF7, IRAK1, NFKB1*	*n. d*.	*DDX58, MAP3K1, TRADD*	*n. d*.
miR-491-3p	Up	*APOBEC3G*	*n. d*.	*n. d*.	*n. d*.
	Down	*CCL5, CD40, CD86, CTSS, CXCL10, CXCL11, IFNA1, IFNA2, IFNB1, IRAK1, IRF7, MAP3K7, MAPK1, MAPK14, MYD88, NFKB1, NFKBIA, TLR3, TLR8, TLR9*	*CASP1, NOD2, PSTPIP1*,	*AZI2, CASP8, CXCL10, DAK, DDX58, FADD, MAP3K1, MAPK14, NFKB1, NFKBIA, PIN1, TRADD, TRAF6, TRIM25*	*IFNA1, IFNA2, IFNAR1, IFNB1, IL15*

Genes were grouped to TLR, NOD-like, RIG-I-like and type I IFN signaling pathway and responsive genes. n. d., not detected.

### The Effect of miRNA Inhibition on Prf, GrzmB, and IFNγ Expression in NK-92 Cells

At last, we additionally analyzed the expression of Prf, GrzmB and IFNγ intracellular expression in NK-92 cells transfected with miR-27b, miR-199b, miR-369-3p and miR-491-3p inhibitors by flow cytometry (**Figure 3**). The results showed that only perforin expression was significantly upregulated in NK-92 cells transfected with miR-369-3p or miR-491-3p inhibitors, when compared to negative control (**Figures 3A, D**). In the case of miR-369-3p inhibitor, both the percentage of cells expressing perforin, and perforin MFI was markedly elevated, while in the case of miR-491-3p inhibitor we only noted upregulation of the percentage of Prf-positive cells. There were no differences in the percentage of cells or MFI when GrzmB (**Figures 3B, E**) and IFNγ (**Figures 3C, F**) expression was analyzed.

## Discussion

In this report, we show that NK cells isolated from patients suffering from severe and/or recurrent HSV infection had dysregulated miRNA expression, what may alter antiviral response of these cells and, as a consequence, escalate HSV infection.

First of all, the results of miRNA expression profiling showed that patients suffering from severe and/or recurrent HSV infection had significantly upregulated expression of four miRNAs: hsa-miR-27b, hsa-miR-199b, hsa-miR-369-3p and hsa-miR-491-3p, when compared to healthy control subjects. None of these miRNAs has been previously reported to play an important function in NK cells, although miR-27a, a close homolog to miR-27b (20/21 nucleotide identity), was shown to repress the cytotoxic capacity of NK cells (24). However, miR-27b, miR-199b, and miR-491-3p had been previously associated with viral infections, also caused by Herpesviruses. miR-27b was up-regulated in human CMV-infected glioma cells (27), and both miR-27a and miR-27b have antiviral activity against murine CMV (28). miR-199b was significantly downregulated in Kaposi’s sarcoma specimens (29), while miR-491 was one of the several miRNAs which expression levels were altered by HCV infection in hepatoma cells (30). There is limited data about the role of miR-369-3p in immune response with most of the research concerning its impact in different types of cancer (31–33).

Selective inhibition of miR-27b, miR-199b, miR-369-3p and miR-491-3p expression in NK-92 cells resulted in down- or upregulation of a number of genes associated with antiviral response. These genes might be involved in the modulation of 4 signaling pathways: Toll-like receptor (TLR) signaling (34), NOD-like receptor signaling (35), the retinoic acid-inducible gene I (RIG-I)-like receptors (RLRs) signaling (36) and type I IFN signaling and response (37). To our surprise, among analyzed miRNAs, miR-27b, miR-199b and miR-491-3p inhibition resulted in upregulation of only four genes in total, while the use of all four inhibitors resulted in downregulation of 36 genes altogether. Both miR-27b and miR-199b inhibition resulted in upregulation of the mitochondrial innate immune adaptor (*MAVS*) gene, while miR-27b inhibitor caused upregulation of two more genes: *MAP2K3* and *TLR7*. MAVS (also known as IPS-1/VISA/Cardif) interacts with the retinoic acid-inducible gene I (RIG-I)-like receptors (RLRs) such as RIG-I and melanoma differentiation-associated gene 5 (MDA-5), which recognize viral RNA, and activate the expression of type I interferons and IFN-stimulated genes (ISGs) (38, 39). Although, HSV is a double-strand DNA virus, it has evolved multiple mechanisms to evade the host innate immune response, including MAVS-mediated response and early ISGs production (40, 41). Moreover, the absence of MAVS alters normal NK cell development and maturation (42). Thus, we believe that MAVS might be a key protein associated with alteration of NK immune response against HSV caused by upregulation of miR-27b and miR-199b in our patients suffering from severe and/or recurrent HSV infection. However, it should be also pointed out that although we did not noticed upregulation of type I interferon signaling and response genes following MAVS upregulation, it is possible the time point of harvesting NK-92 cells after stimulation was too short to observe MAVS-mediated gene expression stimulation.

miR-27b inhibitor significantly upregulated *TLR7* gene and *MAP2K3* gene, which mediate downstream signaling of TLR receptors. TLR7 and TLR8 recognize the single-stranded RNAs (ssRNAs) found in many viruses, leading to IFN-α production in virus-infected macrophages and dendritic cells (43, 44). It has been shown, though, that infection of human corneal epithelial cell (HCEC) line by HSV-1, which is a dsDNA virus, can lead to upregulation of TLR7 expression level and proinflammatory cytokine and IFNs production (45). On the other hand, MAP2K3 engagement in the activation of p38-STAT1 pathway and activation of a subset of ISGs in antigen-presenting cells during HIV-1 infection was previously reported to facilitates expansion of the infection (46). MAP2K3/P38 MAPK signaling pathway plays also essential role in coxsackievirus B3 infection, favoring viral progeny release from infected cells (47). MAP2K3 has been also shown to be inhibited by miR-21 (47), which promotes NK cell proliferation (48). Taken together, it is possible that upregulation of miR-27b expression in NK cells of our patients promotes HSV infection. Moreover, MAP2K3/P38 MAPK signaling pathway involvement in HSV infection needs further investigation.

miR-491-3p inhibition resulted in upregulation of *APOBEC3G* (*A3G*) gene expression, which is a type I IFN-responsive gene. A3G is a cytidine deaminase, an enzyme that belongs to a larger group that can edit nucleic acids. A3G is as a well-known interaction partner of the HIV-encoded Viral Infectivity Factor (Vif) protein, which counteract the antiviral activity of A3G proteins (49). However, increased expression of *A3G* in NK cell lines profoundly alters the coding transcriptome of NK cells observed under hypoxic stress (50). Since both hypoxia and chronic infections are stress factors for NK cells, we might assume that A3G could play an important role in NK cell functions during prolonged HSV infection. Latent HCMV infection has already been shown to downregulate APOBEC3G in CD34^+^ progenitor cells (51).

To our surprise, a large number of antiviral response-mediating genes were downregulated in NK-92 cells by miRNA inhibitors. In particular, miR-27b inhibition resulted in downregulation of *MAP3K7, MAPK1* and *MAPK14* genes, coding TLRs signaling factors. Similarly, miR-369-3p inhibition resulted in cathepsin S-coding gene *CTSS*, which is crucial in regulating TLR9 signaling (52) and *CCL3, CCL5, IRF7, IRAK1 and NFKB1* genes, encoding TLRs signaling factors. Both inhibitors also downregulated several RIG-I-like receptors signaling genes: *AZI2, CASP1, MA3K1, PIN1* and *TRADD* (in case of miR-27b) and, in case of miR-199b, *DDX58, MAP3K1* and *TRADD* and type I IFN signaling genes: *IFNAR1, IL15*. Moreover, the latter two miRNA inhibitors, specific for miR-199b and miR-491, also contributed to downregulation of a number genes coding for TLR signaling factors: *TLR8, TLR9, CTSS, IRF7, MAP3K1, MAP3K7, MAPK1, MAPK14*, NFKB1, NFKBIA, or TLR responsive genes: *CD40, CD86, CXCL11, IFNA1, IFNA2*, and *IFNB1*. miR-491-3p inhibitor additionally downregulated *CLL5, IRAK1, MYD88* and *TLR3* gene expression. Both miRs inhibitors mediated downregulation of genes belonging to NOD-like receptor signaling: *CASP1, NOD2* (both miRs), *PSTPIP1* (miR-491-3p); and RIG-I-like receptor signaling and responsive genes: *AZI2, CASP8, CXCL10, DAK, DDX58M* (RIG-I), *FADD, IFNA1, IFNA2, IFNB1, PIN, TRADD and TRIM25*. Moreover, they also downregulated type I IFN signaling- associated genes: *IFNA1, IFNA2, IFNAR1, IFNB1* and *IL15*. Since a number of genes downregulated by miR-199b and miR-491-3p highly exceed a number of upregulated genes, we think that upregulated expression of miR-199b and miR-491-3p observed in our patients might not be directly associated with NK cell function impairment, leading to observed prolonged HSV infection, but provides a type of a compensation mechanism enhancing defective NK cell function. However, this issue requires further studies.

Flow cytometry analysis of Prf, GrzmB and IFNγ intracellular expression in NK-92 cells transfected with miR-27b, miR-199b, miR-369-3p and miR-491-3p inhibitors revealed that Prf expression (the percentage of perforin-expressing cells or perforin MFI) was upregulated by miR-369-3p and miR-491-3p inhibitors, while the expression of GrzmB and IFNγ remained unchanged. Previously, Prf has been shown to be posttranscriptionally regulated by miR-30e (25) and miR-150 (23). GrzmB expression is targeted by miR-378 (53), while miR-146a plays a negative role in IFN-γ production by human NK cells (54). Thus, this is the first report indicating that miR-369-3p and miR-491-3p play a role in negative regulation of perforin expression.

In conclusion, our study suggests a novel mechanism which may promote recurrence and severity of HSV infection based on the miRNAs-dependent posttranscriptional regulation of genes taking part in antiviral response of human NK cells.

## Data Availability Statement

The raw data supporting the conclusions of this article will be made available by the authors, without undue reservation.

## Ethics Statement

The studies involving human participants were reviewed and approved by Bioethical Committee of the Jagiellonian University. Written informed consent to participate in this study was provided by the participants’ legal guardian/next of kin.

## Author Contributions

ML and MSi designed the research. ML, ED, AK, KW, MR-Z, and MSu performed the experiments. ML, ED, and MR-Z analyzed the data, generated figures and tables. ASz, AP-N, and MSi recruited patients and control group. ML wrote the manuscript. MSi and MSa discussed and edited the manuscript. All authors contributed to the article and approved the submitted version.

## Funding

This work was supported by the National Science Centre of Poland (NCN) (grant number 2013/09/D/NZ2/01660).

## Conflict of Interest

The authors declare that the research was conducted in the absence of any commercial or financial relationships that could be construed as a potential conflict of interest.
