# Supplementary material for: miRNA Regulation of NK Cells Antiviral Response in Children With Severe and/or Recurrent Herpes Simplex Virus Infections
Source: Front Immunol. 2021 Jan 25;11:589866. doi: 10.3389/fimmu.2020.589866 (PMC7931645; doi:10.3389/fimmu.2020.589866)
Supplement: Supplementary file 1 [file DataSheet_1.docx]

Supplementary Material

# Supplementary Figures and Tables

## Supplementary Figures

**Supplementary Figure S1. FACS sorting strategy of NK-92 cells transfected with anti-miR inhibitor.** Living cells were gated on the basis of FSC/SSC parameters (A). The histogram presenting untransfected cells (left peak) and cells transfected with FAM-conjugated anti-miR- inhibitor (right peak) (B).
