# Supplementary figures and images for: miRNA Regulation of NK Cells Antiviral Response in Children With Severe and/or Recurrent Herpes Simplex Virus Infections
Source: Front Immunol. 2021 Jan 25;11:589866. doi: 10.3389/fimmu.2020.589866 (PMC7931645; doi:10.3389/fimmu.2020.589866)

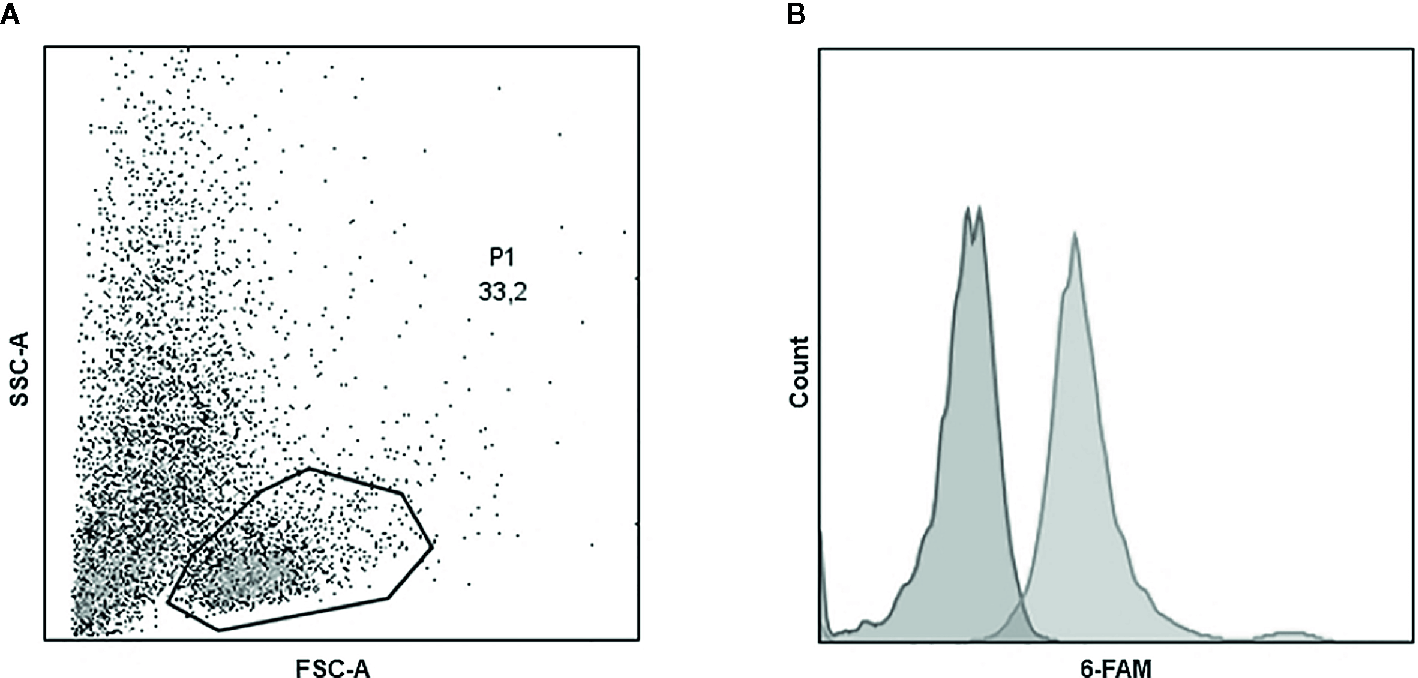

Supplement: Supplementary file 2 [file Image_1.tif]
